# Correction: Elbedwehy et al. Super Effective Removal of Toxic Metals Water Pollutants Using Multi Functionalized Polyacrylonitrile and Arabic Gum Grafts. *Polymers* 2019, *11*, 1938

**DOI:** 10.3390/polym18070783

**Published:** 2026-03-24

**Authors:** Ahmed M. Elbedwehy, Ali M. Abou-Elanwar, Abdelrahman O. Ezzat, Ayman M. Atta

**Affiliations:** 1 Nanotechnology Center, Mansoura University, Mansoura 35516, Egypt; 2 National Research Centre, Dokki, Giza 12622, Egypt; science_ali87@yahoo.com; 3 Chemistry Department, College of Science, King Saud University, P.O. Box 2455, Riyadh 11451, Saudi Arabia; ao_ezzat@yahoo.com

## Error in Figure

In the original publication [1], there was a mistake in Figure 1 as published. The authors regret that an unintentional visual misalignment was introduced in the spectral presentation during figure preparation. We sincerely apologize for this oversight and any confusion it may have caused. The corrected Figure 1 is provided below. The authors confirm that the scientific conclusions are unaffected. This correction was approved by the Academic Editor, and the original publication has been updated.

## Figures and Tables

**Figure 1:**
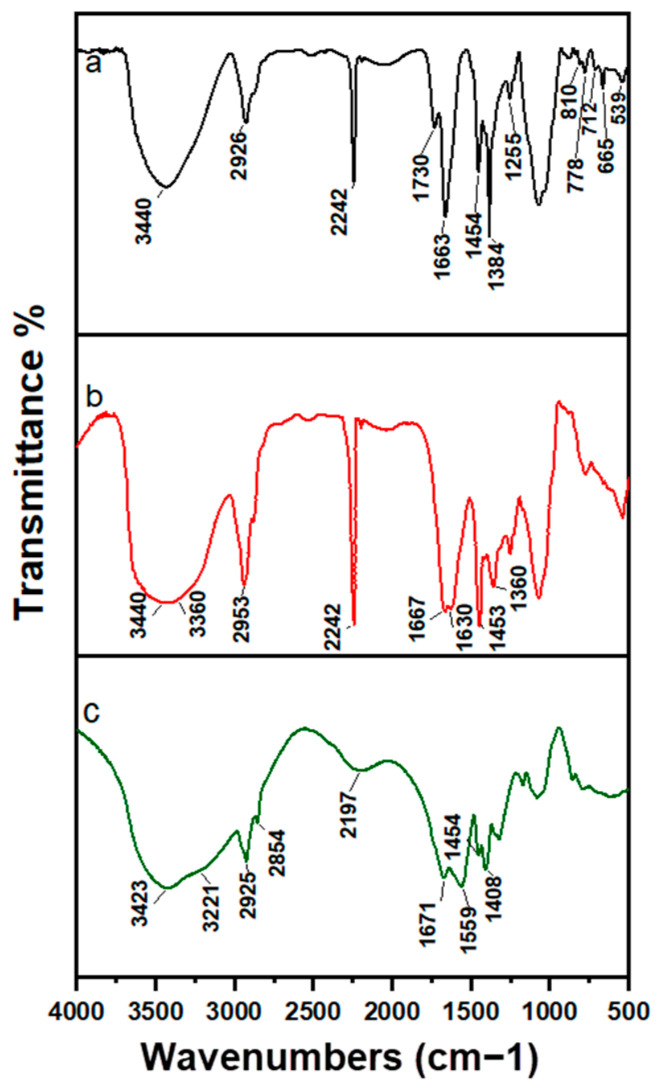
FTIR spectra of (**a**) PAN–*g*–AG, (**b**) hydrazine-modified PAN–*g*–AG, and (**c**) Second modification of PAN–*g*–AG with sodium hydroxide.
